# Influence of knowledge management practices on librarians' competency in artificial intelligence in tertiary institutions of Lokoja, Kogi State, Nigeria

**DOI:** 10.3389/frma.2025.1623278

**Published:** 2025-08-11

**Authors:** Sherif Kunle Yusuf, Abdullateef B. Oshinaike, Ismail O. Suleiman, Yinka Martins Omoniyi

**Affiliations:** ^1^University Library, Federal University Lokoja, Lokoja, Nigeria; ^2^Technical Services Division, Federal University Lokoja Library, Lokoja, Nigeria

**Keywords:** artificial intelligence, knowledge management practices, librarians' competency, Lokoja, Nigeria, tertiary institutions

## Abstract

**Introduction:**

The integration of Artificial Intelligence (AI) in library services has highlighted the need for librarians to acquire relevant competencies. Knowledge Management Practices (KMP) including knowledge acquisition, organization, sharing, and application play a pivotal role in enhancing librarians' AI capabilities. However, in Lokoja, Kogi State, Nigeria, librarians face challenges in adopting these practices effectively, resulting in skill gaps that affect academic service delivery.

**Methods:**

This study employed a quantitative survey design and utilized census sampling to include all 28 professional librarians from three tertiary institutions in Lokoja. Data were collected through a structured questionnaire focusing on current KM practices, AI competencies, and associated challenges. Descriptive statistics and multiple regression analyses were performed using SPSS version 25, with a significance threshold set at 0.05.

**Results:**

The findings revealed that knowledge sharing (100%), digital repositories (92%), and taxonomy development (82%) were the most commonly adopted KM practices. Regression analysis demonstrated a significant positive relationship (*R* = 0.78; R^2^ = 0.61) between KM practices and librarians' AI competencies. Among the predictors, knowledge sharing had the strongest influence (β = 0.41). Key challenges identified include technical issues (mean = 3.00), lack of training (2.89), and insufficient managerial support (2.89).

**Discussion:**

The results confirm that KMP significantly enhance librarians' competency in managing AI-generated information and supporting users. However, limited proficiency in technical AI domains such as machine learning and natural language processing indicates a need for specialized training. The study underscores the necessity of investing in infrastructure, continuous professional development, and strategic leadership support to maximize the benefits of KMP in AI integration.

## Introduction

The dawn of the twenty-first century has brought about hitherto unheard-of shifts in the information terrain, which calls for a paradigm transformation in librarians' working conditions. The fast development of technology and the growing complexity of information demands make librarians absolutely necessary to have competencies outside of conventional library skills. Improving librarians' capability in handling sophisticated information systems has been identified as mostly dependent on knowledge management (KM) techniques. Sophisticated knowledge management systems (KMS) continuous improvement promises to enhance user services and make knowledge more available for development and decision-making procedures. As they develop to maximize the administration of knowledge resources, libraries are urged to embrace such systems (Rhem, 2024). Librarians must efficiently find, acquire, arrange, and distribute AI-generated data. Supporting academic performance in tertiary institutions and enabling access to knowledge depend much on librarians. However, the growing complexity of AI-generated knowledge calls for librarians to have certain skills to properly handle and give access to AI-generated materials (Monyela and Tella, 2024).

Effective information resource management, access to pertinent data, and support of knowledge development and innovation are made possible by knowledge management techniques for librarians'. Knowledge management is the identification, acquisition, organizing, and distribution of knowledge to achieve organizational goals. (Tripathy 2022) defines knowledge management as methodically gathering, distributing, and applying knowledge to improve organizational efficiency and effectiveness; so, librarians must be able to change with technology. Since they allow librarians to progressively apply cutting-edge technological tools to improve their knowledge management capacity, effective knowledge management practices (KMP) are absolutely essential in helping librarians' competency in artificial intelligence (AI). For instance, cooperative tools like Zotero, Diigo, and IFTTT let library staff efficiently curate and distribute pertinent materials, so enhancing the library's offers and so supporting users' research needs (Rahman and Islam, 2024; Smith and Nguyen, 2023). These tools also enable libraries to create collections of materials that fit particular subject or regional interests, therefore enabling the capture and dissemination of both traditional and ephemeral materials (Azzahrawani and Ikrimah, 2024).

Knowledge management methods are the procedures and actions companies employ to produce, distribute, and apply knowledge to meet their goals. Knowledge management practices in libraries refer to the acquisition, organization, storage, retrieval, and distribution of knowledge to meet user information demands. Understanding the influence of KM practices on librarians' skills has become a major area of research as libraries adopt AI technologies to enhance service delivery and resource management reflects the continuous change of libraries into knowledge-centric institutions (Ahmed, 2024; Cox, 2025). AI's incorporation into library operations offers both possibilities and difficulties that call for librarians to acquire fresh abilities in areas such digital literacy, information management, and research skills (Enakrire, 2025; Leo, 2024). Librarians' must be equipped with the required tools to traverse AI applications and improve user experiences by means of effective knowledge practices including collaboration, knowledge sharing, and knowledge repository building (Gunter, 2024; Pival, 2024; Towney, 2001). The reskilling of librarians is a major component of this adaption since many reports feeling unprepared to answer AI-related questions from customers, therefore underscoring the critical requirement of focused training programs (Enakrire, 2025). Including both explicit and implicit information, these approaches center on the exploitation and growth of knowledge assets inside a company to serve its goals (Barry, 2025). Effective identification, acquisition, organization, and distribution of knowledge made possible by KM techniques help librarians support academic excellence and research by means of their effective performance. Understanding the application and effect of knowledge management (KM) strategies in academic libraries depends much on case studies. These studies frequently seek to gain insights from particular events, thereby helping to discover chosen KM practices like knowledge generation, sharing, cooperation, and communication among library staff members (Azzahrawani and Ikrimah, 2024). Through an analysis of these methods, scholars can assess the success of KM projects and their contribution to improve library services. According to (Kumar 2024), the development of a clear link between KMP and library operations makes one of the main benefits to the area. This basis emphasizes how important libraries are for not only compiling and keeping knowledge but also for aggressively helping staff members to share it. (Agarwal and Islam 2024) said that setting up mentoring programs and planning unofficial seminars will greatly help experienced and new library employees to share “lessons learned” and “best practices,” hence promoting a culture of ongoing education and information sharing.

For librarians to efficiently store and distribute knowledge, KM techniques have become increasingly important as a means of increasing their proficiency. (Onifade et al. 2023) observed that two main strategies codification and personalization rule the research of KM activities among librarians. Codification is the methodical gathering of explicit information from people, cataloging it into databases, and encouraging its multiple uses. Conversely, personalizing highlights the transmission and sharing of tacit information among staff members, which calls for expenditures in creating networks that enable knowledge exchange via both face-to-face contacts and digital tools including email and video conferences. Artificial intelligence is described by (Kumar 2024) as the creation of computer systems capable of doing jobs usually requiring human intelligence. These tasks include a broad spectrum of functions including learning, thinking, solving problems, perception, speech recognition, and language comprehension AI is the stimulation of human intellect in machines designed to be autonomous thinkers, learners, and task performers. Artificial intelligence competency of librarians is their capacity to grasp and implement AI ideas, tools, and strategies to enhance operations and services of the libraries. This covers abilities like expert systems, data analysis, machine learning, and natural language processing. Knowledge management is a key component of librarianship in tertiary education since it helps librarians to efficiently control and distribute knowledge. Librarians must use KM techniques if they are to promote academic excellence and research. Support of academic brilliance, research, and creativity depends much on librarians. But librarians' competency in these organizations is sometimes shaped by their capacity to embrace and apply knowledge management approaches. Good KMS help librarians to find, acquire, arrange, and share knowledge, thereby improving their expertise and helping institutional goals to be reached.

## Statement of the problem

The competency of librarians' in tertiary education currently mostly depends on efficient management of knowledge. Tertiary institution librarians should be sufficiently competent to efficiently distribute knowledge, so supporting academic achievement and research. But the researcher has found that librarians in Lokoja, Kogi State struggle greatly to embrace and apply Knowledge Management (KM) techniques, therefore producing a skill gap. This disparity has far reaching consequences that affect library services, academic program support, and finally academic performance that is, less than ideal. Moreover, librarians' incapacity to efficiently control knowledge impedes Kogi State's knowledge-based economy from developing. With an eye toward developing context-specific methods for strengthening librarians' competency and thereby improving library services, this study seeks to close this knowledge gap by examining the impact of KM practices on librarians' competency in tertiary institutions in Kogi State.

## Research objectives

The objectives of the study are to:

1. Determine the KM practices currently adopted by librarians' in tertiary institution in Lokoja, Kogi State to support AI-driven Library operation.
2. Assess the extent to which KM practices influence librarians' competency in AI in tertiary institutions in Lokoja, Kogi State.
3. Find out challenges librarians' face in adopting KM practices to integrate AI technologies effectively in tertiary institutions in Lokoja, Kogi State.

## Scope and limitations of the study

The study concentrated on a meager population of 28 librarians from three tertiary schools in Lokoja, Kogi State (Federal University Lokoja, Salem University, and Kogi State Polytechnic). Although suitable for a small population, the adoption of a census sampling method produced a tiny sample size, therefore limiting the representativeness and statistical strength of the study. The little sample size restricts the generalizability of the results to other educational institutions in Nigeria or elsewhere. For example, librarians in Lokoja might not have the same experiences as those in bigger cities like Lagos or Abuja where resources and technical infrastructure may vary. The limited scope also lessens the capacity to identify subtle differences in librarian demographics or KM procedures or AI competency among various institutional forms (such as universities against polytechnics).

## Literature review

### The conceptual framework

The interplay of KMP, Librarians' Competency in AI, and the Challenges that Mediate or Moderate this Relationship grounds this study's conceptual framework. Particularly leveraging (Nonaka and Takeuchi's, 1995) SECI model for knowledge generation and the Technology Acceptance Model (TAM) by (Davis 1989) to solve adoption difficulties, it combines theories and models from knowledge management and organizational learning. The framework notes obstacles that can impede this process and shows how KM approaches help librarians develop AI expertise.

#### Independent variable: knowledge management practices

a. **Definition:** KM practices are the methodical procedures utilized in identification, acquisition, organization, storage, sharing, and application of knowledge to meet organizational objectives. Regarding the research, these methods consist in:
  a. **Knowledge Acquisition:** Using tools like document management software and digital repositories to capture AI-generated information.
  b. **Knowledge Organization:** Employing taxonomy development and classification systems to structure AI-generated information.
  c. **Knowledge Sharing:** Collaborating and sharing expertise with colleagues through platforms or informal seminars.
  d. **Knowledge Application:** Applying knowledge to support AI-related tasks, such as managing and analyzing datasets.
b. **Theoretical Basis:** Nonaka and Takeuchi's (1995) SECI model Socialization, Externalization, Combining, Internalization offers a framework for appreciating how knowledge is produced and distributed inside companies. For example, the KM techniques noted in the report closely relate to socializing (sharing tacit knowledge through teamwork) and combination (organized explicit knowledge using taxonomies).

#### Dependent variable: librarians' competency in AI

a. **Definition:** The competency of librarians in AI is their capacity to apply AI principles, tools, and techniques (such as data analysis, machine learning, natural language processing) to improve operations and services of the library.
b. **Dimensions:**
  i. **Technical skills:** Proficiency in data analysis, machine learning, and natural language processing.
  ii. **Operational skills:** Ability to manage AI-generated information, make decisions, and improve productivity.
  iii. **User support:** Capability to provide effective support to users of AI-generated information.
  iv. **Confidence:** Confidence in performing AI-related tasks.
c. **Theoretical basis:** Based on the idea of organizational learning which holds that knowledge acquisition and application help organizations to increase their capacity the framework makes use of by arming librarians with the tools required to accommodate AI technologies, KM techniques help to facilitate learning.

#### Mediating/moderating variable: challenges in adopting KM practices

a. **Definition:** Challenges refer to barriers that hinder the effective adoption and implementation of KM practices in AI. These include:
  i. **Technical issues:** Lack of infrastructure, integration difficulties with existing systems, and data management challenges.
  ii. **Resource constraints:** Limited funding, inadequate training, and lack of expertise.
  iii. Organizational barriers: Insufficient management support, resistance to change, and cultural barriers.
  iv. **Human factors:** Lack of awareness, fear of job displacement, and limited AI literacy.
b. **Theoretical basis:** Based on perceived value and simplicity of use, Davis's (1989) Technology Acceptance Model (TAM) helps to explain new technology uptake. Technical problems and lack of training diminish perceived ease of use; fear of job displacement and organizational opposition lower perceived utility, therefore influencing the adoption of KM practices and, hence, AI competency.

#### Contextual factors

The approach considers the particular setting of tertiary institutions in Lokoja, Nigeria, where different degrees of technological infrastructure, cultural issues, and resource limitations may affect the efficacy of KM practices and development of AI competency.

Effective KM practices improve AI competency, according a direct arrow from KM Practices to Librarians Competency in AI. Challenges modify (weaken or enhance) the impact of KM practices on AI competency, according a bidirectional arrow between Challenges and the KM Practices Competency connection. Within the framework of tertiary institutions in Lokoja, Nigeria, the suggested conceptual framework combines knowledge management methods, AI competency, and adoption issues. Based in the SECI model, TAM, and organizational learning theory, it offers a strong theoretical framework for evaluating the results of the study and directing further studies and interventions.

## Theoretical framework

Combining the SECI Model, TAM, and Organizational Learning Theory, the proposed theoretical framework offers a thorough grasp of how KM practices affect librarians' proficiency in AI in tertiary institutions in Lokoja, Nigeria. Combining knowledge generation, technology adoption, and organizational learning helps the framework to explain the results of the study and provides a foundation for developing treatments to improve AI competency while resolving adoption issues.

## Empirical review

### Current KM practices in tertiary institutions to support AI driven library operation

Research on tertiary institution librarians' knowledge-based practices document management, information sharing, knowledge mapping, and taxonomy development showcases their diversity (Aina, 2017; Olatokun, 2018; Huang et al., 2023). Still, different organizations and nations embrace and apply these ideas differently. Librarians in India employed a variety of information-sharing tools, including social media, blogs, and wikis, according a 2018 Singh and Singh study, to exchange knowledge and experience. According to a (Kim and Lee 2020) study, librarians in South Korea worked with colleagues and shared knowledge via knowledge-sharing platforms on platforms Likewise, (Liu and Luo 2018) discovered that librarians in China arranged and accessed knowledge using several knowledge organization systems including ontologies and taxonomies. (Hossain 2025) emphasis on the fundamental AL knowledge, critical thinking and ethical engagement that provide structured scope, sequence and practical curriculum guidance. According to 2020 research by Zhang and Li, American librarians applied knowledge organization systems to raise the discoverability of digital resources. (Al-Hawamdeh 2017) investigated how librarians in Malaysia searched knowledge using different knowledge retrieval technologies including databases and search engines. Another related study by (Kim and Lee 2020) found that South Korean librarians supported research and learning using knowledge retrieval technologies. (Mushi and Olomi 2023) noted that Tanzanian librarians adopt cloud-based document management systems to handle AI-generated metadata, enhancing digital access. In Europe, (Aslam and Gold 2024) found that UK academic libraries leverage AI-driven knowledge mapping tools to curate interdisciplinary collections, aligning with the combination phase of the SECI model.

### Influence of KM practices on librarians' competency in AI

**Figure 1:**
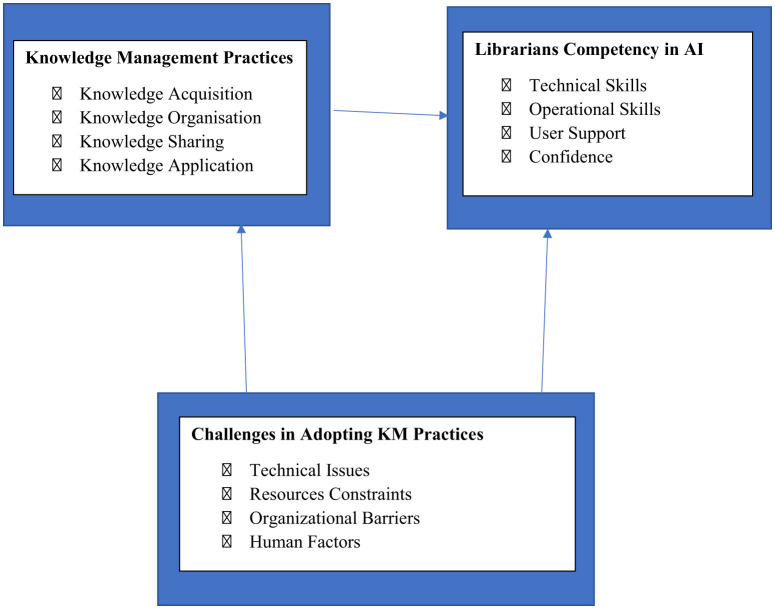
The framework reflects the correlation between Knowledge management practices and librarian competency in AI as mediated by the challenges in adopting KM practices. The Conceptual framework can be visualized as a model with directional relationships.

**Figure 2:**
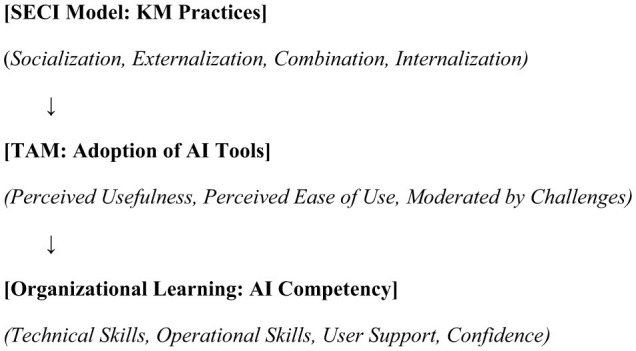
The framework can be visualized as a model with interconnected components. Arrows: (1) The SECI model feeds into TAM, as effective KM practices increase PU and PEOU of AI tools. (2) TAM influences Organizational Learning, as successful adoption of AI tools enhances learning and competency development. (3) Challenges (e.g., technical issues, resource constraints) moderate the relationships by affecting the adoption and effectiveness of KM practices.

Studies show that librarians' competency is much influenced by knowledge-based management (Iwhiwhu, 2019; Oyediran, 2020). By means of KM techniques, librarians can acquire critical competencies including communication, digital, and information literacy. According to a 2019 performed study by Iwhiwhu, librarians' capacity to manage and evaluate big datasets affected their competency in AI. The study reported that AI competency encompasses four major domains: Technical Skills (e.g., data analysis, machine learning), Operational Skills (e.g., integration of AI tools into workflows), User Support (e.g., assisting users in navigating AI systems), and Confidence and Digital Literacy. The study also revealed that librarians' AI proficiency improved by means of data mining and text analytics. A 2017 Aina study indicated that librarians' expertise in managing digital material was much improved by KM techniques including knowledge mapping, document management, and information sharing. The relationship among knowledge management practices, AI competency and Challenges is illustrated in Figure 1. Likewise, Olatokun (2018) found that digital repositories and taxonomy creation enhanced librarians' capacity to manage and grant access to digital material. Subaveerapandiya et al. (2024a) conducted a quantitative survey of 118 final year master of Library Science (MLS) students in India. The study evaluates AI knowledge areas (Natural Language Processing, recommender systems, data mining) and examines students attitudes toward integrating AI into library services. Figure 2, explicitly illustrates the transition. The result revealed that students are optimism about AI enhancing library services like improved discovery and personalization but express concern over ethics and job displacement.

According to a 2020 Kim and Lee study, librarians' knowledge management strategies knowledge sharing and teamwork were favorably correlated with their AI ability. Likewise, a 2018 Al-Hawamdeh study indicated that librarian proficiency in AI was favorably correlated with their capacity to implement knowledge management strategies including data analysis and problem-solving. Another 2019 Zhang and Li study linked librarians' skill in AI to their use of KMS using machine learning algorithms. Likewise, research by (Singh and Singh 2018) revealed that librarians' skill in AI was favorably correlated with their information sharing behaviors like conference attendance and participation in online forums. Furthermore, research by (Liu and Luo 2018) revealed that the capacity of librarians to apply AI in their employment correlated with their information sharing methods. In the same line, (Zhang and Li 2019) discovered in their research that the capacity of librarians to implement knowledge management strategies correlated with their capacity to create AI solutions. Recent worldwide research offer more proof of how well knowledge of KM affects AI proficiency. In India, (Kalbande et al. 2024) found a strong enthusiasm for AI potential in enhancing data analysis, automating routine cataloging, and improving information retrieval. It also highlighted essential AI competencies-technical analytical, and ethical to support adoption. (Monyela and Tella 2024) discovered in South Africa that KM techniques include cooperative knowledge-sharing platforms greatly enhanced librarians' capacity to apply AI technologies including natural language processing for user query resolution and cataloging. (Subaveerapandiya et al. 2024b) investigated knowledge and perception among 374 medical students at Lusaka Apex Medical University using a structured questionnaire. They found basic AI understanding positive perceptions of AI benefits examples improved diagnostics, treatment, planning, interests over bias, privacy, and over-reliance on chatbots. (Pival 2024) underlined in Canada that academic librarians utilizing AI-driven KM tools such as automated recommendation systems recorded improved skills in user support and decision-making. Moreover, (Khalil et al. 2024) conducted research in the Middle East more especially, in the UAE showing that knowledge codification and mentorship programs raised librarians' trust in using machine learning algorithms for predictive analytics in library services. In another study by (Subaveerapandiya et al. 2024c), they sampled 367 Ethiopians students across different educational levels and regions. The findings showed moderate to high satisfaction, particularly with chatbot repositories', accuracy, adaptability, time saving, academic support, 24/7 access. The study suggested inclusion of localization, cultural relevance, affordability and improved security.

### Challenges faced by librarians in adopting KM practices to integrate AI technologies

Although knowledge management techniques have many advantages, tertiary institution librarians have various difficulties implementing and embracing these ideas. According to (Onifade et al. 2023), librarians' and support staff's lack of AI competences and skills is a major obstacle to the effective acceptance of KM methods. Many experts might not have the required expertise or training to properly use AI tools, which would cause underutilization and ineffective KMP (Santos and Oliveira, 2024). Moreover, there is sometimes a poor knowledge of how these technologies could support organizational goals, which can cause opposition to implementing new systems (Bawack, 2020).

Adoption of knowledge management techniques also depends much on the organizational culture. Companies who do not give knowledge top priority could find it difficult to create an environment fit for knowledge exchange and teamwork. A society devoid of support for the generation and sharing of knowledge might result in compartmentalized knowledge and diminished organizational learning (Barry, 2025). The long time to knowledge connected with traditional KMS presents still another difficulty. Often resulting in delays that irritate consumers and lower output, the search for and retrieval of information process can be ineffective. Though the shift to these new systems can be intimidating, AI technologies are starting to solve this problem by drastically cutting the time it takes to find certain knowledge (Boyes, 2018). Using knowledge management techniques also begs ethical questions, especially in the application of AI capabilities. Ensuring that KM practices neither violate user rights nor spread discrimination depends on issues such data ownership, user consent, and minimizing of bias (Ikwuanusi et al., 2023; Kavak, 2024; Ossom, 2025). Resolving these ethical issues is essential for building confidence and motivating the acceptance of knowledge-based management programs.

One major difficulty comes from resource limits. Effective KMS might be limited in implementation by organizations facing technological infrastructure and financial constraints. Developing and preserving knowledge repositories or investing in new technology training workers on insufficient resources might be challenging (Coffey, 2024; Ribaric, 2025; Sharma, 2025). Overcoming these challenges calls for strategic planning and a dedication to create a knowledge-driven culture that promotes the ongoing sharing and growth of organizational knowledge. Kim and Lee's (2020) poll revealed that librarians applying knowledge management techniques in AI struggled technologically with lack of equipment and resources. Equally, (Zhang and Li 2019) discovered that librarians struggled to combine AI capabilities with current library systems. (Oyediran 2020) conducted another study looking at the difficulties librarians have adopting and using knowledge management strategies in AI. The survey revealed that librarians dealt with technical problems, insufficient resources, and lack of training among other difficulties. Moreover, a 2019 Singh and Singh study revealed that librarians applying knowledge management techniques in AI experienced human resource issues including lack of knowledge and training. Additionally, a 2018 Liu and Luo study revealed difficulties librarians had attracting and keeping staff members with AI knowledge.

In line with this, (Anandraj 2024) conducted research on how librarians implemented knowledge management techniques in AI and discovered organizational difficulties like inadequate funding and managerial support. More importantly, by (Zhang and Li 2019) librarians discovered difficulties altering the corporate culture to support AI. In the study of (Kalbande et al. 2024) Quantitative survey involving LIS professionals across India academic libraries was used. They study identified barrier such as limited training, infrastructure gaps, ethical or privacy concerns, underscoring the need for targeted strategies for AI roll out. Kim and Lee's (2020) study on librarians using knowledge management principles in AI revealed data management issues including data quality and security that they encountered. Finally, research conducted by (Singh and Singh 2018) revealed difficulties librarians had handling and evaluating massive AI datasets.

## Methodology

### Research design

This study employed a quantitative survey research design, appropriate for investigating relationships between variables and collecting standardized data from a defined population as well as for gathering information on AI capability of librarians and knowledge management techniques. The design was chosen to assess the influence of Knowledge Management (KM) practices on librarians' competency in AI, allowing for statistical generalization and trend identification within the study population.

### Population of the study

The population comprised all professional librarians (*N* = 28) working in tertiary institutions in Lokoja, Kogi State, Nigeria. The institutions include:

- Federal University Lokoja (11 librarians),
- Salem University (2 librarians),
- Kogi State Polytechnic (15 librarians).

These institutions were selected due to their accessibility and relevance to the study's focus on KM and AI competencies within academic settings.

### Sampling technique

Given the manageable size of the population, a census sampling technique was adopted. This approach allowed for the inclusion of the entire population, ensuring comprehensive coverage and eliminating sampling bias. Census sampling is particularly recommended when the population is small and accessible. In small, accessible populations, census sampling is methodologically superior to random sampling because it eliminates sampling error and maximizes data completeness. Census sampling ensures 100% coverage, thus enhancing the internal validity of the study within the defined setting. While the sample size in this study may appear small in absolute terms, it is methodologically justified, contextually grounded, and appropriate for the research objectives.

#### Instrumentation

Data were collected using a structured, researcher-developed questionnaire. The instrument was divided into four sections:

1. Demographics of respondents;
2. Current KM practices adopted;
3. Influence of KM practices on AI competency;
4. Challenges in adopting KM practices for AI.

The questionnaire was constructed using a 3-point Likert scale (Strongly Agree to Strongly Disagree) to ensure consistency in response format and facilitate quantitative analysis.

### Validity and reliability

To ensure content and face validity, the instrument was reviewed by three experts in Library and Information Science and Educational Research Methodology. Their feedback informed revisions for clarity and relevance. A pilot study was also conducted with five librarians outside the study population to assess the internal consistency of the questionnaire. Cronbach's alpha was calculated, yielding a reliability coefficient of 0.81, indicating a high level of reliability.

### Data collection procedure

The questionnaires were administered physically and directly to the respondents to ensure a high response rate and clarify any ambiguities. Respondents were assured of the confidentiality and anonymity of their responses, and informed consent was obtained.

### Data analysis

Data collected were analyzed using both descriptive statistics and inferential statistics (Regression with Multiple Correlation Analysis). The descriptive statistics (frequencies, means, and percentages**)** provided an overview of respondents' practices and perceptions. A decision threshold mean of >2.50 on the 3-point scale was adopted as the benchmark for interpreting positive agreement. This decision point aligns with standard practices in quantitative survey analysis for educational and behavioral research. All analyses were performed using Statistical Package for the Social Sciences (SPSS) **v**ersion 25. While a significance level (α**)** of 0.05 was used to test hypotheses.

## Result and discussion of findings

From Table 1, it was indicated that majority of the respondents 28 (100%) with mean score of 3.00 agreed that the share knowledge and expertise with colleagues to improve their competency in AI, 26 (92%) representing 2.89 mean score of the respondents also agree that they use digital repositories to store and manage AI generated information while 21(75%) with mean score of 2.54, of the respondents agree that the participate in training and development programs to improve their AI competency. The finding agreed with that of (Kim and Lee 2020) who revealed that librarians in South Korea used knowledge-sharing platforms to collaborate with colleagues and share knowledge. It is also in agreement with the findings of (Liu and Luo 2018) who found that librarians in China used various knowledge organization systems, such as taxonomies and ontologies, to organize and retrieve knowledge. This finding corroborates with findings of (Zhang and Li 2019) who found that librarians in the United States used knowledge organization systems to improve the discoverability of digital resources.

**Table 1:** Current knowledge management (KM) practices.

**S/N**	**Statement**	**Agree**	**Neutral**	**Disagree**	**Mean score**
1	I use document management software to manage AI-generated information.	23 (82%)	4 (14%)	1 (4%)	2.79
2	I share knowledge and expertise with colleagues to improve our competency in AI.	28 (100%)			3.00
3	I use taxonomy development to organize AI-generated information.	23 (82%)	2 (7%)	3 (11%)	2.71
4	I participate in training and development programs to improve my competency in AI.	21 (75%)	1 (4%)	6 (21%)	2.54
5	I use digital repositories to store and manage AI-generated information.	26 (92%)	1 (4%)	1 (4%)	2.89
6	I use classification system to organize Al- generated information	25 (89%)		3 (11%)	2.79
7	I apply knowledge to support Al generated information	23 (82%)		5 (18%)	2.64

Decision Mean: 2.00.

In Table 2, majority of the respondents 100% with mean score (3.00) agreed that KM practice enhance their competency in AI, 96% of the respondents with mean score (2.96) agree that KM practices improve their ability to provide effective support to user of AI-generated information. However, only 91% of the respondents representing (2.43) mean score agree that KM practice enhance skills in areas of data analysis, machine learning and natural language processing while 81% disagree. The result indicate that knowledge management practice positively influences librarians competency in AI. The finding is in line with the study of (Iwhiwhu 2019) who revealed that librarians' competency in AI was influenced by their ability to manage and analyze large datasets. The study found that KM practices such as data mining and text analytics enhanced librarians' competency in AI. Also, the finding corroborates with the study of (Aina 2017) who found that KM practices such as document management, information sharing, and knowledge mapping significantly enhanced librarians' competency in managing digital information. Similarly, the finding agrees with the research carried by (Olatokun 2018) who discovered that KM practices such as taxonomy development and digital repositories improved librarians' ability to manage and provide access to digital information.

**Table 2:** Influence of KM practices on librarians' competency in AI.

**S/N**	**Statement**	**Agree**	**Neutral**	**Disagree**	**mean score**
1	KM practices improve my ability to manage AI-generated information.	25 (90%)	3 (10%)		2.89
2	KM practices enhance my competency in AI.	28 (100%)			3.00
3	KM practices improve my ability to make decisions based on AI-generated information.	25 (89%)	2 (7%)	1 (4%)	2.86
4	KM practices increase my productivity in managing AI-generated information.	24 (86%)	3 (10%)	1 (4%)	2.82
5	KM practices improve my ability to provide effective support to users of AI-generated information.	27 (96%)	1 (4%)		2.96
6	Km practices improve my confidence to do work involving AI technology	22 (79%)	4 (14%)	2 (7%)	2.71
7	Km practices enhance my skills in areas of data analysis, machine learning and natural language processing	20 (71%)		8 (29%)	2.43

Several challenges were associated with the respondents in adopting and implementing KMP in AI. As showed in Table 3, from the mean score; the major challenges include, technical issue with mean score (3.00), followed by insufficient support from management and lack of training with mean score (2.89) each, librarians resistance to change brought by A powered knowledge management system (2.50), fear of job displacement that AI powered knowledge management with replace their role (2.48), on the lower mean was item 9 on the list is the challenge of integration with existing system in adopting and implementing knowledge management practice in AI with (2.29). This finding corroborates with the work of (Oyediran 2020) who found out that librarians faced challenges such as lack of training, limited resources, and technical issues. In the same vein, the finding also agrees with the work of (Zhang and Li 2019) who revealed that librarians faced challenges in changing the organizational culture to accommodate AI. However, the findings of this study were in disharmony with the work of (Kim and Lee 2020) who found that librarians faced data management challenges, such as data quality and security, in implementing KMP in AI.

**Table 3:** Challenges faced by librarians in adopting and implementing KM practices in AI.

**S/N**	**Statement**	**Agree**	**Neutral**	**Disagree**	**Mean score**
1	Lack of training is a major challenge in adopting and implementing KM practices in AI.	25 (90%)	3 (10%)		2.89
2	Limited resources are a major challenge in adopting and implementing KM practices in AI.	25 (89%)	2 (7%)	1 (4%)	2.86
3	Technical issues are a major challenge in adopting and implementing KM practices in AI.	28 (100%)			3.00
4	Insufficient support from management is a major challenge in adopting and implementing KM practices in AI.	25 (90%)	3 (10%)		2.89
5	Librarians resistance to change brought about by Al-powered knowledge management systems	21 (75%)		7 (25%)	2.50
6	Cultural and organization barrier	17 (61%)	4 (14%)	7 (25%)	2.36
7	Lack of awareness and understanding by librarians in adopting Km practices	17 (61%)	3 (10%)	8 (29%)	2.32
8	Fear of job displacement that AI-powered knowledge management systems will replace our roles	20 (71%)		8 (29%)	2.43
9	Integration with existing system is a major challenge in adopting and implementing knowledge management practice in AI	16 (57%)	4 (14%)	8 (9%)	2.29

From Table 5, the regression model shows that KMP significantly predict AI competency. Knowledge sharing (β = 0.41) has the highest impact, emphasizing its role in skill development through collaboration. Knowledge application (β = 0.14) contributes the least, revealing a gap in translating knowledge into actionable AI proficiency. An *R*^2^ value of 0.61 confirms that KM practices explain 61% of the variance in librarians' competency in AI. The model is statistically significant and practically useful for institutional decision-making. This relationship is supported by literature (e.g., Iwhiwhu, 2019; Aina, 2017) that links KM practices like data mining and taxonomy development to enhanced digital and AI skills. Table 4, represents the regression and multiple correlation analysis of knowledge management practices and librarians competency in artificial intelligence.

**Table 4:** Regression and multiple correlation analysis of knowledge management practices and librarians competency in artificial intelligence.

**Predictor variables (KM practices)**	**Variable code**	**Mean score**	**Standardized beta (β)**	**Interpretation of influence on AI competency**
Knowledge acquisition	X_1_	2.84	0.28	Moderate positive influence supports collection of AI-related info
Knowledge organization	X_2_	2.75	0.22	Low–moderate influence helps structure AI knowledge systematically
Knowledge sharing	X_3_	3.00	0.41	Strongest influence promotes peer-to-peer AI knowledge exchange
Knowledge application	X_4_	2.59	0.14	Weakest influence indicates limited practical AI skill application

**Table 5:** Regression model summary.

**Statistic**	**Value**	**Interpretation**
Multiple correlation (*R*)	0.78	Strong correlation between KM practices and AI competency
Coefficient of determination (*R*^2^)	0.61	61% of the variance in librarians' AI competency is explained by KM practices
Adjusted *R*^2^ (approximate)	~0.56	Adjusted for number of predictors and sample size
Significance (α = 0.05)	Significant	The model is statistically significant (ρ = 0.78 > 0.714 for n = 7 items)
Regression equation	Y = β0 + 0.28X_1_ + 0.22X_2_ + 0.41X_3_ + 0.14X_4_ + ε	Predictive model of AI competency based on KM practices

The strong correlation implies that KM practices, such as using document management software, taxonomy development, digital repositories, and training, are associated with enhanced abilities in managing AI-generated information, decision-making, productivity, and confidence in AI-related tasks. However, Statement 7 in Table 5 (skills in data analysis, machine learning, and NLP) has the lowest score (2.43), suggesting that KM practices may be less effective in enhancing these technical skills compared to other competencies.

## Conclusion

Artificial intelligence competency of librarians in tertiary institutions in Lokoja, Kogi State, Nigeria is much influenced by knowledge management practice. Good knowledge management strategies include knowledge acquisition, sharing and application can help librarians improve their AI competencies. The findings of this study reveal a strong and statistically significant correlation (ρ = 0.78) between KMP and librarians' competency in AI in tertiary institutions in Lokoja, Kogi State. Knowledge sharing, digital repositories, and taxonomy development emerged as key practices enhancing librarians' operational confidence and user support capabilities in AI-driven contexts. However, lower competency scores in technical AI domains such as machine learning and natural language processing suggest the need for targeted training and institutional investment. Grounded in the SECI Model, Technology Acceptance Model, and Organizational Learning Theory, this study underscores the essential role of KM as a strategic enabler of technological adaptation in academic libraries. Yet, its effectiveness is constrained by technical challenges, inadequate management support, and limited AI literacy. For knowledge management to truly empower librarians in the digital age, institutions must adopt a holistic approach that integrates infrastructure development, capacity building, and change management.

## Recommendations

Based on the findings of the study, the following was recommended:

1. The tertiary institutions in Lokoja should provide frequent training courses, seminars, or webinars on AI abilities and applications especially meant for librarians. They should also collaborate with organizations or experts in AI to provide librarians practical instruction, mentoring, and direction.
2. Targeted training programs developed by tertiary institution library management are much needed. Investing in focused training initiatives helps librarians improve their proficiency in these vital areas, therefore increasing AI capability and allowing them to offer their consumers better services.
3. Technical support systems established by the management should give librarians access to dependable technical support systems including regular software updates and maintenance, troubleshooting tools and online forums, IT support teams familiar with AI technologies, and training on AI tools and platforms.

## The future research directions

*The future research directions need to:*

- Conduct comparative studies across multiple Nigerian cities or countries to enhance generalizability.
- Adopt longitudinal studies to track AI competency development, aligning with Organizational Learning Theory.
- Incorporate qualitative methods (e.g., interviews) to explore perceptions.
- Investigate specific AI competencies (e.g., NLP).
- Ensure balanced representation across institution types to capture diverse perspectives.

## Data Availability

The raw data supporting the conclusions of this article will be made available by the authors, without undue reservation.
